# Health care access and satisfaction in Judean and Samarian communities: opportunities for improving care

**DOI:** 10.1186/s13584-018-0227-y

**Published:** 2018-09-03

**Authors:** Ephraim Shapiro, Avi Zigdon, Rachel Nissanholtz-Gannot

**Affiliations:** 1 Department of Health Systems Management, Ariel University, 4 Kiryat Hamada, 40700 Ariel, Israel; 2 Smokler Center for Health Policy Research, Myers-JDC-Brookdale Institute, Jerusalem, Israel

**Keywords:** Health care access, Patient satisfaction, Judea, Samara, West bank, Israel

## Abstract

**Background:**

There are distinctive potential barriers to optimal health care in Judea and Samaria because of access and satisfaction levels, including obstacles such as its isolation and health care capacity. However, there is a lack of research focusing on health care for the Jewish communities in this region, often referred to as the West Bank.

**Research questions:**

What is the level of health care access and satisfaction for Israelis living in the Jewish communities in Judea and Samaria?

How do these results compare to parallel results for Israelis in general?

How do these results vary by subgroups, in particular by location?

**Methods:**

Two hundred fourty six residents of Judea and Samaria in six diverse, Jewish communities were surveyed, with a 76% response rate. Descriptive analyses were performed for all variables. Bivariate analyses for access and satisfaction measures were performed by key demographic variables. Comparisons were also made with Israelis in general (the vast majority of whom do not live in Judea or Samaria), by comparing our survey results to the results of 2016 Myers-JDC-Brookdale Institute national satisfaction survey. Our survey questions were based on this national survey, tested and used for several cohorts.

**Results:**

Of those surveyed, 14% decided to forego treatment because of the distance, although only 3% declined treatment because of cost. There was a diversity of results in terms of satisfaction measures, although in no categories were even half of respondents very satisfied; results ranged from 7% very satisfied with health care system overall to 47% very satisfied with their family physician’s attitude. Variations were found by community with local council communities generally, but not always, having the highest satisfaction. Compared to Israelis in general, Israeli residents of Judea and Samaria reported generally lower satisfaction, including 9% fewer being very satisfied with the health plan overall and 10% fewer being very satisfied with referrals. However, 7% more had confidence in getting the best treatment.

**Conclusions:**

Access to care involves more than just coverage. Health care system problems among Israelis living in Judea and Samaria include not just quantity, but quality of services offered. There is a need for improvement not only in health care resources, but also in the level of access and satisfaction in this region.

## Background

Access to health care is a key factor that can affect health system effectiveness and health inequity [1, 2]. Inadequate access can affect health care quality for the individual and raise the burden of disease for society [3]. The term “access” can involve more than just availability of care, but also other elements of receiving health care such as a feeling of connection with providers or language concordance [1, 4].

These latter measures overlap with patient satisfaction, which can affect both clinical outcomes for patients as well as economic outcomes such as patient retention and malpractice claims [5]. Level of satisfaction with health care services can vary among populations within a country, leading to health inequity [2].

Patient satisfaction and access can be considered important elements of patient centered care with a relationship existing between them [2, 6], and with each potentially influencing the other [5, 7, 8]. This approach to care is a topic growing in interest and importance, and has been shown to be related to better quality of care, although its impact can vary [9, 10].

In addition, patient satisfaction and access can be related to each other in both directions. Accessibility affects the satisfaction of the patient with his treatment [6], and studies have found a positive correlation between patient satisfaction and greater health care continuity and accessibility [11, 12].

OECD countries, in general, are increasingly concerned with access and patient satisfaction, and Israel is no different [13]. Since 2009, the Israeli Ministry of Health has undertaken initiatives to reduce health inequity [14, 15]. Among the program’s goals is removing obstacles to appropriate access to health services, yet it has not focused on Judea and Samaria, commonly known as the West Bank.

Judea and Samaria is a large region, with about 5% of the population of the State of Israel living there – almost 400,000 residents [16]. Israel has four health plans, called “kupot”, equivalent in many ways to Health Maintenance Organizations (HMOs) that cover and coordinate patient care for residents of Israel, although non-citizens may need to pay additional fees to obtain coverage [17]. However, there may be inadequate access to care for residents of Judea and Samaria despite Israeli universal health care. There are distinctive challenges to adequate health care service provision in this region that are important to understand and try to overcome. Most of the Jewish communities in Judea and Samaria are relatively small cities and villages, which are dispersed over a wide area, and typically have limited transportation access [16]. There is also evidence of a clinician shortage in the region [18, 19].

While there have been a number of studies investigating access to care and satisfaction for the Arab population in this area [20–23], to our knowledge none have focused on the Jewish communities of Judea and Samaria. There have been studies on health care access and patient satisfaction of Israelis in general, but they have either omitted Judea and Samaria or had limited success in recruiting respondents from this area [13, 24, 25]. This study aims to contribute towards filling that gap, with important implications for improving population health.

Specifically, this study examines:

1. What is the level of health care access and satisfaction for Israelis living in Jewish communities in Judea and Samaria?
2. How do these results compare to parallel results for Israelis in general?
3. How do these results vary by subgroups, in particular by type of community?

We hypothesize that there are distinctive barriers to health care access and satisfaction in these communities, with important implications for achieving appropriate health service use patterns and potentially improving health in this region.

## Methods

Israeli residents of selected Jewish communities in Judea and Samaria were surveyed. To increase degree of representativeness, a diversity of location types was included. The target population included:

- Two of the largest cities in the region: Ariel and Modiin Illit
- Two local councils: Alfei Menashe and Karnei Shomron
- Two regional councils: Mateh Binyamin and Megillot, which include a number of small villages.

The study population was randomly sampled based on a phone list within each community where possible, supplemented by a face-to-face convenience sample. A maximum of one person per household was selected. Both landline and cellphone numbers were used. About 90% of respondents were reached by telephone, with the remainder interviewed face to face. They were initially attempted to be reached by landline and if not successful an attempt made to reach them by cellphone. The proportion of landline reached by landline, cellphone, or in-person was not tracked.

Surveys were administered by trained students at Ariel University in Samaria. Interviews were conducted in Hebrew. Of the 322 households that were approached, the final sample included 246 residents over the age of 20 living in the six target communities responded, 76.4% of the households. Data were collected from October 2015 through January 2016. No identifying information was collected. The study was approved by the Ariel University ethics board.

The questionnaire was based on a survey instrument developed and used by the Myers-JDC Brookdale Institute (MJBI) for a national satisfaction survey typically conducted every 2–3 years [24]. It consisted of about 70 questions with sections that focused on access to health care, patient satisfaction, health services utilization, branch and staff hours, health status, and demographics. Respondents were asked to rate their level of satisfaction for a variety of services: very satisfied, satisfied, not so satisfied or not satisfied.

Descriptive analyses were performed for all variables. Bivariate analyses for key access and satisfaction measures were performed for the six target locations as well as for other demographic variables; chi-square statistics were produced to test for significance of differences. Comparisons were also made with results, where available, for Israelis in general, using data from the 2016 MJBI survey; Z-ratios were produced to test for significance of the differences between proportions in two samples.

## Results

### Demographics

Descriptive statistics for the sample’s demographic characteristics can be found in Table 1. A third of respondents were between ages 20–34, with only a tenth above age 65. The sample was 63% female. A large majority of respondents were born in Israel, with Hebrew being their primary language. Less than half of the sample had a university education. About a quarter of the sample had monthly household income below 7000 NIS (about $2000), a low amount as the average household income is 18,671 NIS. Respondents were divided approximately evenly among the self-identified religious categories of secular, traditional, religious, and Haredi (often called ultra-orthodox). The proportion of the sample living in the six target communities ranged from 7% living in Megillot to 29% living in Modiin Illit.

**Table 1:** Demographics of the Sample

Variable	Category	Percent (N = 246)
Community	Ariel	21
	Modiin Illit	29
	Alfei Menashe	14
	Karnei Shomron	16
	Mateh Binyamin	14
	Megillot	7
Age	20–34	33
	35–64	57
	65+	10
Gender	Female	63
Marital Status	Married/Partner	77
	Widowed/Divorced	9
	Never Married	13
Children in Household	0–1	24
	2–4	49
	5+	27
Education	University	45
	Post-High School Seminary(religious study)	24
	High School or below	31
Income	0–7000	32
	7000–14,000	41
	> 14,000	27
Religiosity	Haredi	23
	Religious	25
	Traditional	24
	Secular	27
Country of Birth	Israel	76
	Former Soviet Union	10
	Other	14

Note: Sum of responses for each category may not equal 100% because of rounding

### Health care access and satisfaction

Health status and insurance statistics can be found in Table 2. All respondents had basic health insurance, like all Israeli citizens, with 86% purchasing supplemental insurance from the health fund; 20% also purchased private insurance. In terms of health status, 47% of respondents rated their health as “very good”, and 14% reported that their health as “not so good”, “fair” or “poor”. About 20% of the sample had at least one chronic disease, and a similar percentage suffered some type of psychological distress in the past year.

**Table 2:** Health Status and Insurance Related Measures

Variable	Category	Percent (N = 246)
Health Status	Very Good	47
	Good	39
	Not so good/Fair/Poor	14
Chronic Disease	Yes	20
Psychological Distress	Yes	20
Health Fund	Clalit	49
	Leumit	18
	Maccabi	24
	Meuchedet	9
Private health insurance	Yes	20
Supplementary health insurance	Yes	86

Full access and satisfaction findings are listed in Tables 3 and 4. Results indicate that most residents are able to see a doctor, although not always in a timely manner, and are often less than fully satisfied with a number of aspects of the visit; 92% of respondents visited a family doctor in the past year and 54% consulted a specialist within the last three months. However, almost a quarter of respondents had to wait a month or more for their appointment to see a specialist and another fifth had to wait at least two weeks. About 22% of respondents went without treatment because of the wait time. There was low satisfaction with provider hours, with only 25% being very satisfied with family doctor hours and just 8% being very satisfied with specialist hours.

**Table 3:** Health Care Access measures

Measure	Percent (N = 246)
Family Doctor visit in the last year	92
Specialist visit in last 3 months	54
Wait time for specialist of a month or more	23
Did not get care because of payments	3
Did not get care because of wait time	22
Did not get care because of distance	14
Confidence in getting best treatment if needed	50
Confident in being able to pay if needed	38
Always Received information needed	64

**Table 4:** Patient Satisfaction Measures

Satisfied with:	% Very Satisfied	% Satisfied	Not Satisfied/ Dissatisfied
Family Physician: Professional Level	44	45	12
Family Physician Attitude	47	44	9
Nurse Attitude	37	49	15
Specialist Physician: Professional Level	29	53	19
Ease of Obtaining Referrals	29	38	33
Family Doctor Hours	25	53	22
Specialist Doctor Hours	8	47	45
Office/Administrative Hours	18	59	24
Laboratory Hours	22	47	31
The Health Plan Overall	28	56	17
The Health Care System Overall	7	60	33

In addition to wait time and hours, another important barrier was transportation, as 14% of the sample decided to forego treatment because of the distance. However, only 3% declined treatment because of the cost.

There was a diversity of results in terms of measures of satisfaction, although there were no categories were even 50% of respondents were very satisfied and in several categories a quarter or less of the respondents were very satisfied.

Only 7% of respondents were very satisfied with the health care system overall and a third were not satisfied/dissatisfied. Twenty-eight percent of respondents were very satisfied with their health plan, with another 56% being at least satisfied. Only half of residents said that they were confident in receiving the most effective treatment should they become ill. An even lower percent, 38%, were confident they could afford to get needed care when seriously ill.

### Comparisons to Israelis in general

**Fig. 1:**
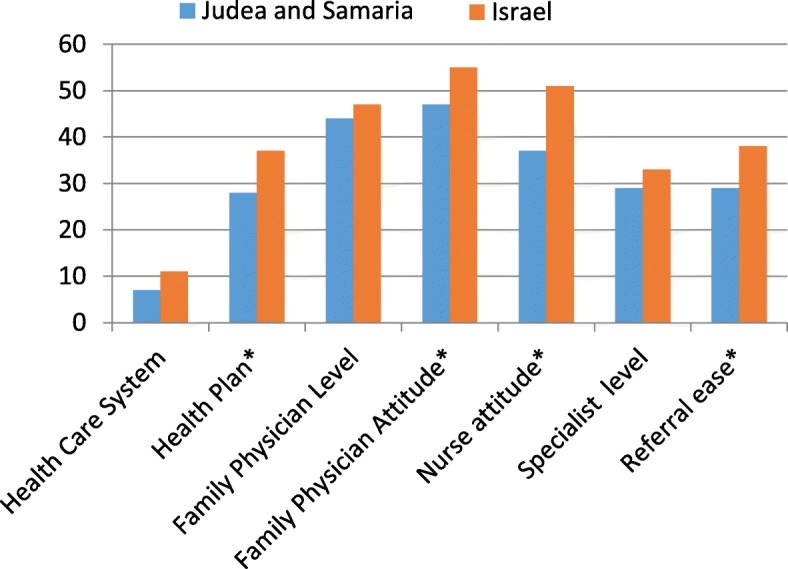
Patient Satisfaction among Israelis in Judea and Samaria vs all Israelis. Percent very satisfied by satisfaction measure. Judea and Samaria *N* = 246 Israel *N* = 2236. * = statistically significant, at least *p* < .05

**Fig. 2:**
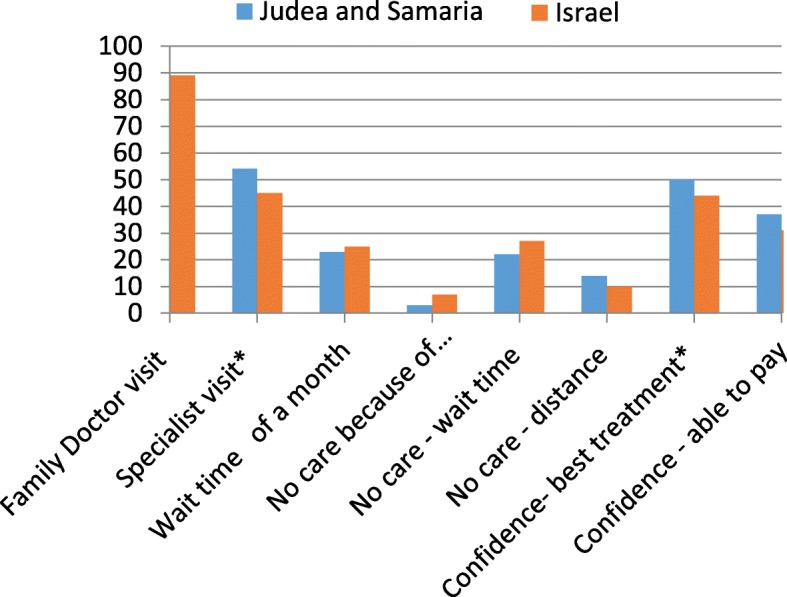
Health Care Utilization and Access among Israelis in Judea and Samaria vs all Israelis. Percent answering yes to each question by access measure. Judea and Samaria N = 246 Israel *N* = 2240. * = statistically significant, at least *p* < .05

We then compared our findings to those from 2016 MJBI satisfaction survey regarding Israelis in general [24]. Results can be found in Figs. 1 and 2. Use of health care services was comparable or slightly higher than for Israelis in general, but Israeli residents of Judea and Samaria reported lower satisfaction in every single category, whether including only those very satisfied or all of those at least satisfied. Differences mentioned below are statistically significant at *p* < .05 unless otherwise noted.

The percentage of those very satisfied with the Israeli health funds was 9 points lower among those residing in Judea and Samaria. No differences were found among individual health funds. In terms of specific aspects of the process of getting care, the percentage of respondents very satisfied with attitudes of family doctors and nurses were 7 and 14 points lower, respectively, than for Israelis in general and satisfaction with referrals was almost 10 points lower.

Despite the lower satisfaction, Israeli residents of Judea and Samaria reported 7 percentage points greater confidence in getting the best treatment. In addition, when compared to Israeli respondents overall, the proportion of respondents living in Judea and Samaria who reported not receiving care because of financial issues was lower and the proportion not receiving care because of the distance was higher, although in both cases only marginally significant.

### Subgroup comparisons

Bivariate analyses were performed for access and satisfaction measures by key demographic categories including age, gender, location, education, income, primary language, marital status, and health fund. The only variable for which there was statistically significant variation for the large majority of measures was location. Results for satisfaction by location can be found in Table 5.

**Table 5:** Patient Satisfaction by Location

Percentage Very Satisfied with:	Cities (Ariel and Modiin Illit)	Local Councils Alfei Menashe and Karnei Shomron)	Regional Village Councils: Mateh Binyamin and Megillot)
N	115	72	50
Family Physician Professional Level	40%	54%	37%
Family Physician Attitude*	39%	63%	44%
Nurse Attitude*	30	54	29
Specialist Physician: Professional Level	25	37	27
Ease of Obtaining Referrals *	26	35	24
Wait Times for Appointments*	16	19	25
Health Plan Overall	27	32	26
The Health Care System*	11	6	0

* *p* < .05

There were a great number of salient differences for satisfaction by type of community, with a diversity of findings; despite the small sample sizes, statistically significant differences were found for the majority of satisfaction measures. The local councils generally had the best results, although not always. For example, cities had the largest proportion of residents who were very satisfied with the health care system overall.

There were fewer statistically significant differences among barriers to care (results not shown in the table), but still some notable findings that were statistically significant, especially in terms of wait time. Analyses were also run for each city individually and some differences found but because of the small sample size for some cities, it was difficult to draw conclusions.

## Discussion

Although Israeli residents of Judea and Samaria were found to regularly utilize the health care system, their level of satisfaction with health services was both suboptimal in itself as well as lower than Israel’s general population, especially in terms of wait times and doctors’ attitudes, an important element of patient-centered care [9]. In addition, there were significant differences among communities for most access and satisfaction measures.

While there have been a number of articles related to the health care access of Arab residents in the region studied [19–23], there have been almost none focusing on the primarily Jewish areas that we researched. One reason for the paucity of literature on this topic may be because it involves a politically sensitive and often controversial geographic area.

The authors believe that the topic is one that warrants attention for a number of reasons. Health is a human right and all of the area’s residents should receive appropriate access to and quality of health care [26]. In addition, findings that improve the health of one group of residents in a region can also benefit other groups in the area. The authors believe the research questions addressed in this study are important ones, regardless of one’s geopolitical opinions, and that the findings make a valuable contribution, with important implications, discussed further below.

Israel has universal health insurance and all the health plans provide a basic and uniform basket of services to all citizens [17]. However, access to care is more than just coverage, often a primary focus of initiatives in some other countries. Given the issues with wait times, referrals, and distance as a barrier, it is likely that health care resources in the area need to be increased. Not all Israeli health plans operate branches in all localities in the region. Moreover, even for branches and clinics that are operating in the region, not all health services are always provided.

There is also a shortage of providers. For example, there are only 3 doctors and 1.5 nurses per 1000 people in Judea and Samaria, both far less than other regions in which Israelis reside [18, 19]. This gap can affect satisfaction level with health services, as patients wait longer to see a doctor, and forego medical care because of wait times.

A review of opening hours for clinics for the four health funds in the region found a diversity of practices. While some clinics had regular evening hours, many either did not have evening hours or when they did, it was for a limited number of days and only until early evening. Although respondents were not asked which specific clinic they used, the limited hours indicate adequate availability of doctors and nurses may be a problem for certain population segments for whom it is difficult to visit providers during working hours. This is consistent with findings, indicating substantial dissatisfaction with doctor hours, especially specialists. Expanded hours of clinics in Judea and Samaria should be considered.

Our findings indicated that problems include not just quantity of services but quality and satisfaction with services offered, indicating a potential need to improve some elements of patient-centered care in the region. Within plans, patients can choose their community-based physicians, both primary and specialist, from physicians affiliated with the plan [17]. Despite this, the majority of respondents are less than very satisfied in almost every category and at levels generally lower than those of Israelis in general, especially attitudes of the clinical staff as well as ease of getting referrals.

A recent study found variations in selected hospital-based procedures by region in Israel including several categories in which utilization for Judea and Samaria [27] was below the Israeli average. The study hypothesized that access to care issues could be a factor in the variations. Our study provides support for this hypothesis, but also that satisfaction may be related to variations.

Interestingly, despite the percentage of respondents who reported low satisfaction with health care services, many reported greater confidence in receiving optimal care and ability to pay for treatment compared to the general population. It is not clear why this is so, although the percentages are still relatively low so this seeming paradox may be an artifact of the percentages for Israelis in general being unexpectedly low.

The issues found in our research appear to be system wide, with virtually no significant differences in outcomes by health plan. However, there were important variations by locality, with the worst results generally found among the regional councils, which contain many small villages. This is consistent with respondent reports of distance being a barrier, as well as literature showing that small and isolated rural villages may have especially limited health care access [28].

### Policy implications

The research conducted has a number of important implications. Findings indicate a need for improvement in the level of access and satisfaction in the region studied because of its distinctive characteristics, especially its isolated location and limited resources, and the barriers to optimal health care that flow from them.

It is likely that health services and other resources in the area need to be increased. An increase in clinical staff could decrease wait times and allow longer hours, both issues identified by respondents. There were variations in access by location, with inadequate access to care especially identified by some smaller localities. As a result, these areas may need special attention, including improved transportation and/or enhanced telemedicine alternatives.

It is not merely access to the health care system that appears to be inadequate, but also the satisfaction with health care services that was found to be problematic. Additional research is needed to fully understand the reasons for this, but the staff in the region may need additional training to better inculcate an ethos of focusing on the patient and/or health services provision may need to be reorganized in the region.

The study also has implications for groups other than Israelis living in the Jewish communities of Judea and Samaria. The health of populations living in proximity to each other can affect each other not only in obvious ways such as increased exposure to infectious diseases, but in less direct ways as well. For example, lessons learned for improving the access and satisfaction in smaller isolated areas can also benefit other populations, both in the same region as well as in Israel generally and in other countries.

### Limitations and additional research

The study is cross-sectional and based on self-reports, with the usual potential limitations to validity and reliability related of subjective responses at a single point in time. Although steps were taken to increase representativeness of the sample, selection was not fully random, with potential for some selection bias resulting. In addition, the proportion reached by landline, cellphone, or in-person was not tracked, so whether or not the method of contact influenced and biased results could not be determined. Further, the direct connection between access and satisfaction to improved health outcomes could not be studied for the target population, although there is evidence linking patient satisfaction with positive health outcomes in general [5].

Although location was the main demographic factor associated with our outcomes during bivariate analyses, performing multivariable analyses when comparing results to national data would be useful to more accurately determine the association of living on Judea and Samaria with these outcomes, independent of other demographic factors.

Additional research in the future should examine this issue, as well as possible causes for the disparities in access and satisfaction. Replication of the study in other communities in the region would also be of value in understanding the generalizability of findings.

## Conclusions

The study’s findings make important contributions to a topic where research is lacking and results suggest initiatives that can be taken to improve access and satisfaction among Israelis living in Judea and Samaria. In addition, future research can build upon this study to help understand how to improve health care for diverse populations in the region. Taking steps to improve access and patient satisfaction can affect the timely and efficient delivery of health care as well as potentially reducing health inequity, compared to Israelis in general.
